# Medium-term survival of patients with mechanical and biological aortic prosthesis at the 6^th^ decade of life

**DOI:** 10.1371/journal.pone.0312408

**Published:** 2024-11-18

**Authors:** Victor Dayan, Juan Andres Montero, Nick Freemantle

**Affiliations:** 1 Centro Cardiovascular Universitario, Hospital de Clinicas, Universidad de la Republica, Montevideo, Uruguay; 2 Institute of Clinical Trials and Methodology, University College London, London, United Kingdom; BSMMU: Bangabandhu Sheikh Mujib Medical University, BANGLADESH

## Abstract

**Objective:**

The best aortic prosthesis type in 60–70 year old patients is not established. Our aim was to evaluate the survival in a National cohort of patients between 60–70 years old who required surgical aortic valve replacement for aortic stenosis (SAVR) with either a mechanical (MP) or bioprosthesis (BP) valve.

**Methods:**

This is a retrospective study using national data from the Ministry of Health. We included all patients between 60 to 70 years old who underwent SAVR for aortic stenosis in Uruguay from 2011 to 2021. The primary outcome was overall survival according to type of prosthesis used stratified by effect modifiers. The independent effects of gender and use of statins were evaluated.

**Results:**

We included 1196 patients (66±3.0 years old; 39.1% female). Mortality was higher for BP (296, 29.9%%) than MP (36, 17.1%; p<0.001). Median follow-up time was 4.5 years (Interquartile range [IQR] 3.4–6.5). The unadjusted incidence rate ratio was higher for BP (Incidence rate ratio [IRR] = 1.43;95%CI: 0.99, 2.14, p = 0.045). The effect of BP on mortality rate was greater in males (IRR = 1.82;95%CI:1.14,2.92. p interaction = 0.08) and patients who were not taking statins (IRR = 1.97;95%CI:1.14,3.41. p interaction = 0.06). The use of BP was an independent predictor of overall survival in male patients (Hazard ratio [HR] = 1.32;95%CI: 1.68, 1.04. p = 0.021) and in patients who were not taking statins (HR = 2.07;95%CI: 1.17, 3.67. p = 0.013).

**Conclusion:**

The use of BP was associated with worse survival in male patients and patients not taking statins. Gender and statins use should contribute to type of prosthesis decision in the 60–69 age group.

## Introduction

Surgical aortic valve replacement (SAVR) or percutaneous prosthetic implantation (TAVI) are effective treatment for severe aortic valve disease. The commonly accepted aortic valve prostheses are either mechanical (MP) or biological (BP). Each has its advantages and disadvantages, with age currently the most accepted clinical variable to define the best option. Mechanical prosthesis are likely to be recommended to younger patients (<50–60 years olds) because of their longer expected durability although they have the disadvantage or requiring anticoagulation, while biological are recommended for older patients (>65 years olds) [1,2]. There is an age range between 60–70 years old in which an individualized approach is considered. For these patients, the only published randomized control trial (RCT) found no differences in survival, albeit with a higher incidence of prosthetic failure and reoperation in BP [3]. A recent meta-analysis of mainly retrospective studies found that MP were associated with improved survival in this age group [4].

Considering the increasing number of TAVI procedures performed in younger patients (<70 years old), it is imperative to confirm the superiority of BP (or not) compared to MP in these patients as well as predictors which may favor mechanical prosthesis. Preliminary evidence suggests that individual patient characteristics may appropriately inform the decision the use of BP or MP in this age range. Gender and use of statins have previously been shown to modify the effectiveness of BP in this age range [5–13].

Considering the risks associated with the use of oral anticoagulation, our hypothesis was that in our region, the use of BP might be associated with better survival. Our aim was to evaluate the survival in a national cohort of patients between 60–70 years old who required SAVR for aortic stenosis with either a MP or BP. Additionally, we explored gender and use of statins as effect modifiers.

## Materials and methods

### Ethical statement

The ethical review board of the Hospital de Clinicas (approval number 6220001. Date 25/3/2022) approved the study and informed consent was not required because of the retrospective nature of the study.

This is a retrospective study using national data from the Fondo Nacional de Recursos (FNR) of the Ministry of Health. The FNR is the national entity which covers 100% of cardiac surgical procedures in Uruguay. Data reporting to the FNR is compulsory to get financial approval for each procedure.

### Patients

We included all 60–70 year old patients who underwent SAVR for aortic stenosis in the 5 surgical centers of Uruguay from 2011 to 2021. Exclusion criteria were: urgent or emergency surgery, endocarditis, moderate aortic stenosis, severe aortic regurgitation, previous cardiac surgery, concomitant mitral or ascending aorta replacement.

Preoperative, intraoperative and immediate postoperative data were extracted from the FNR. Expected mortality was evaluated using Euroscore I. Calculation of Euroscore II was not possible since we included patients before Euroscore II and the registry lacks the required data for its calculation. Vital status and cause of death were extracted from the Department of Epidemiology of the Ministry of Health. Data were accessed on the 01/05/2022.

### Outcomes

The primary outcome was overall survival according to type of prosthesis. Secondary outcomes were cardiovascular survival, postoperative mortality, acute renal failure, postoperative infections, surgical re-exploration due to bleeding, rhythm abnormalities.

### Definitions

All patient baseline characteristics and postoperative outcome definitions were based on definitions used by the FNR.

Operative mortality was defined as mortality during the 30 days after surgery.

Cardiovascular death was defined using the codification provided by the International Classification of Disease (ICD) as death due to I00-I99 as entered in the National Death Certificate.

Acute renal failure was defined as an increase in 50% of creatinine values with respect to baseline (definition used by the FNR).

Postoperative infection was defined as any type of infection (for example: surgical site, upper respiratory, pneumonia, urinary) after the surgical procedure.

Obesity was defined as a body mass index of 30 or higher.

Postoperative pacemaker was defined as the implantation of a pacemaker after surgery.

The use of these definitions is compulsory for the inclusion of patients in the FNR registry.

### Statistics

Continuous data are expressed as mean±SD. Categorical data as absolute value and %.

Preoperative variables were compared among both groups to define potential confounders using chi-squared (for categorical variables) and Student’s t-test (for continuous variables). Potential confounders were then evaluated using univariate Cox regression analysis. Data were missing for NYHA preoperative status in 174 patients. Comparison of baseline characteristics in patients with and without missing NYHA values was performed (S2 Table). Sensitivity analysis was performed through complete case analysis (Cox regression analyses without missing values). Univariate confounding and effect modification were assessed using Mantel-Haenszel stratification and multivariable adjustment was performed using Cox regression. Interaction in the Cox multivariable regression model was evaluated with the likelihood ratio test for the variables found to be modifiers in the univariate analyses. If present, the interaction term was included in the final model. Additional variables with clinical importance not significant in the univariate analyses were also included in the final model.

Long term mortality was evaluated as the mortality rate in which the numerator was number of events (death) and in the denominator was person.days of follow-up. The association of prosthesis type with mortality was evaluated using the incidence rate ratio (IRR).

Survival was described using Kaplan-Meier graphs, and comparison between groups used the log rank test.

Comparison of postoperative outcomes among groups was adjusted for confounders using logistic regression.

Analyses were performed with Stata/IC 16.1 for Mac.

## Results

During the time period of analysis 3944 patients underwent SAVR in Uruguay. Of these, 1196 were within an age range of 60 to 70 years old. Median follow-up time was 4.5 years (Interquartile range [IQR]: 3.4–6.5) (3.2 years in mechanical prosthesis and 4.8 years in bioprosthesis) and follow-up was 100% complete. Mean age of the whole cohort was 66.0±3.0, 39.1% were female patients, mean Euroscore I was 4.85±2.85%. Patients who received a BP were on average older, and with higher prevalence of obesity and CABG as a concomitant procedure. This and additional information is described in Table 1. Expected mortality (EuroSCORE I) was higher in patients who received a BP (4.97±2.83 vs 4.31±2.88, p<0.002). Types of prostheses used are described in S1 Table.

**Table 1:** Basal characteristics of included patients (n = 1196).

	Bioprosthesis (986)	Mechanical (210)	Total	p
Age (SD)	66.5 (2.8)	63.8 (2.9)	66.0 (3.0)	<0.001
Female (%)	393 (38.9)	75 (35.7)	468 (39.1)	0.264
HTN (%)	771 (78.2)	157 (74.8)	928 (77.6)	0.279
Diabetes (%)	237 (24.0)	46 (21.9)	283 (23.7)	0.509
Current smoker (%)	223 (22.6)	51 (24.3)	274 (22.9)	0.601
Obesity (%)	178 (18.1)	26 (12.4)	204 (17.1)	0.047
Creatininemia (mg/dl)	1.06 (0.71)	1.11 (0.97)	1.07 (0.76)	0.335
Use of statins (%)	389 (39.5)	85 (40.5)	474 (39.6)	0.783
Previous MI (%)	17 (1.7)	2 (0.9)	19 (1.6)	0.417
AF (%)	26 (2.6)	5 (2.4)	31 (2.6)	0.832
NYHA III/IV (%)	264 (31.2)	50 (29.0)	314 (30.9)	0.942
LVEF (SD)	57.6 (10.4)	57.8 (11.1)	57.6 (10.5)	0.829
Concomitant CABG (%)	318 (32.3)	53 (25.2)	371 (31.0)	0.046
EuroSCORE (SD)	4.97 (2.83)	4.31 (2.88)	4.85 (2.85)	0.002

AF: Atrial fibrillation; CABG: Coronary artery bypass grafts; HTN: Hypertension; NYHA: New York heart association; LVEF: Left ventricular ejection function; MI: Myocardial infarction.

### Primary outcome

**Fig 1:**
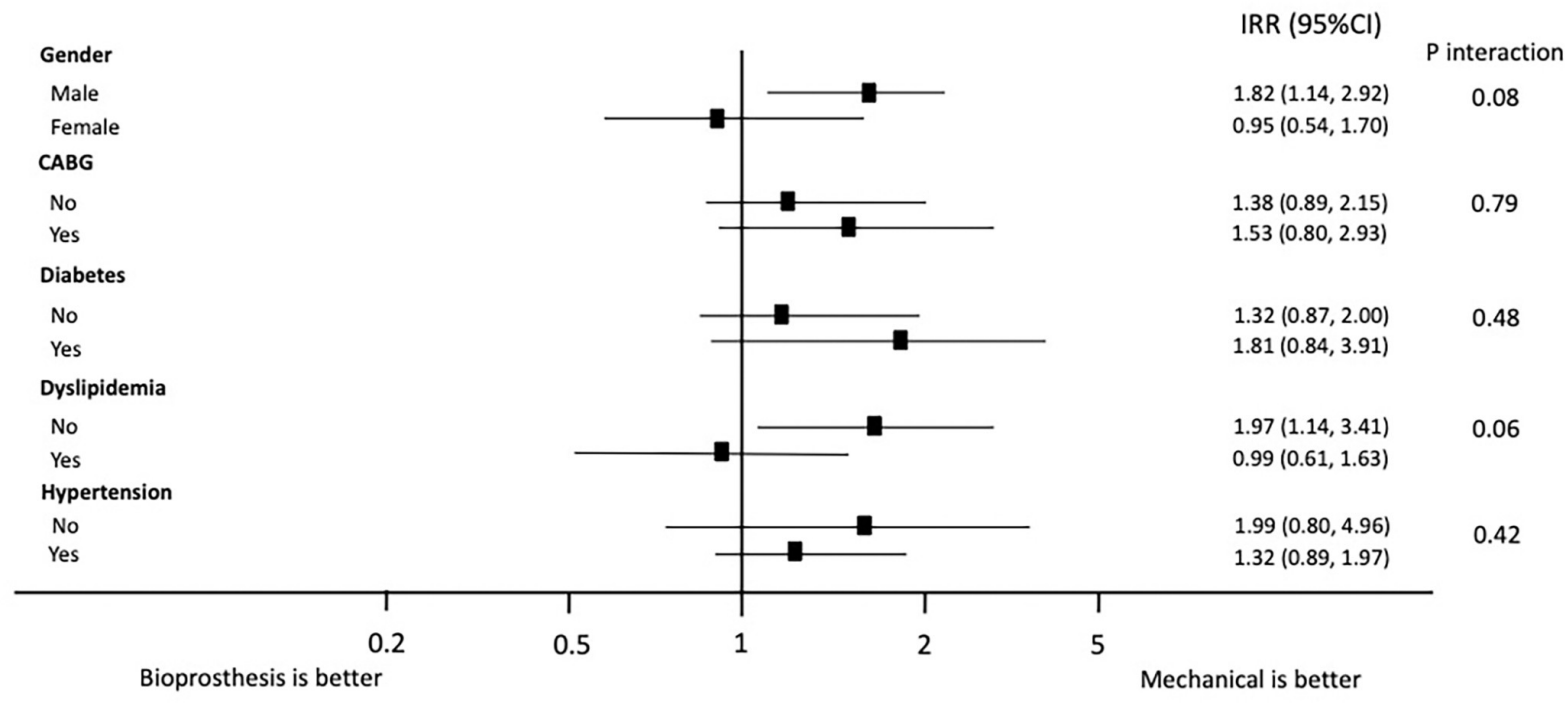
Forest plot for mortality incidence rate ratio for bioprosthesis with adjustment for confounders and stratum-specific associations (n = 1196). CABG: Coronary artery bypass grafts; IRR: Incidence rate ratio.

Mortality was higher for BP(296 patients, 29.9%%) than MP(36 patients, 17.1%; p<0.001). The un-adjusted incidence rate ratio showed an increased risk in patients who received a BP (IRR = 1.43;95%CI: 0.99, 2.14, p = 0.045). Univariate adjustment showed that gender (pint = 0.08) and dyslipidemia (p = 0.06) were potential modifiers of the effect of BP on mortality rate (Fig 1). The use of BP on mortality rate was detrimental in male (IRR = 1.82;95%CI: 1.14, 2.92) or patients without statins (IRR = 1.97;95%CI: 1.14, 3.41) compared with female patients (IRR = 0.95;95% CI: 0.54, 1.70. pint = 0.08) or patients taking statins (IRR = 0.99;95%: 0.61,1.63. pint = 0.06).

**Fig 2:**
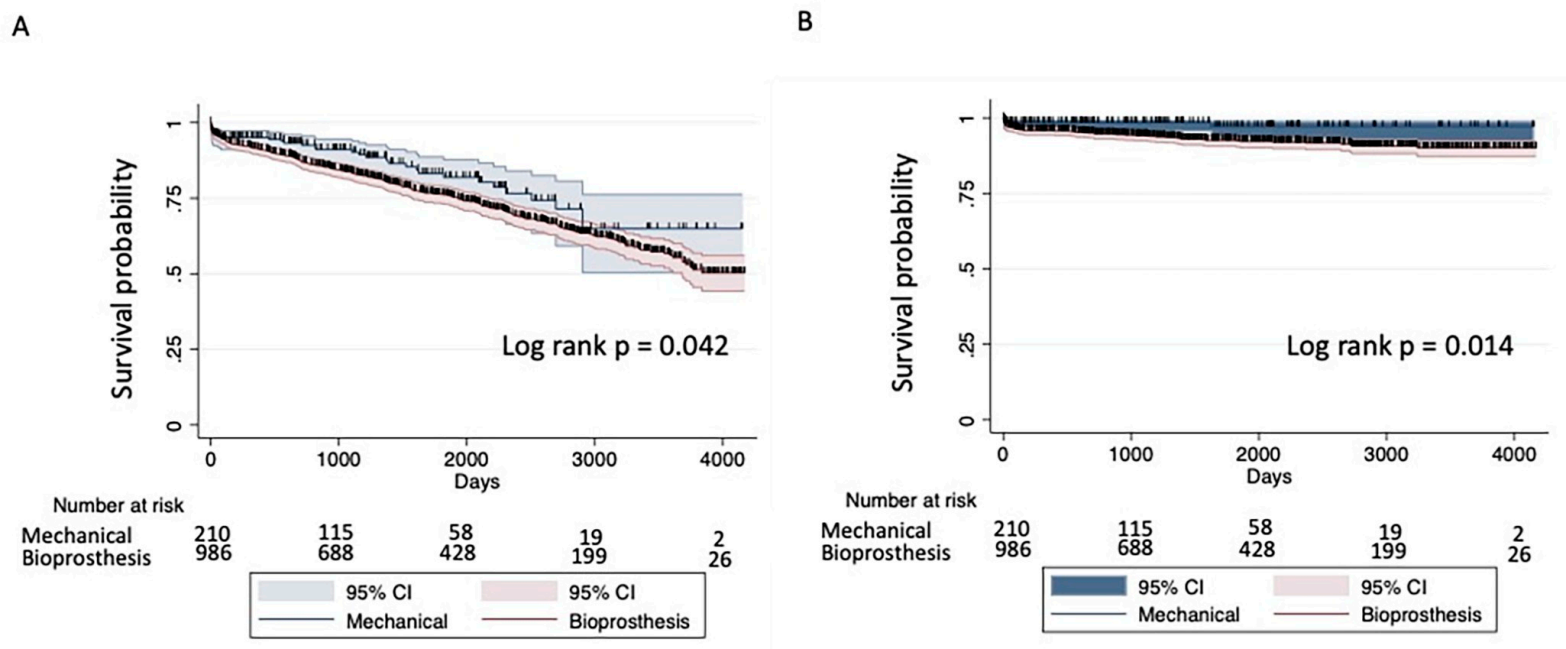
Kaplan-Meier survival curves. A) Overall and B) Cardiovascular survival.

Un-adjusted overall mortality (log rank p = 0.042) and cardiovascular survival (log rank p = 0.014) were lower in patients who received BP (Fig 2).

Univariate predictors of survival are described in S2 Table: age (HR = 1.06;95%CI: 1.02, 1.10. p = 0.002), creatinine (HR = 1.16; 95%CI: 1.06, 1.27. p = 0.001), EuroSCORE I (HR = 1.10; 95%CI: 1.08, 1.13. p<0.001), BP (HR = 1.46; 95%CI: 1.01, 2.11. p = 0.042), use of statins (HR = 1.28;95%CI: 1.03, 1.60. p = 0.026), diabetes (HR = 1.39;95%CI: 1.09, 1.77. p = 0.008), hypertension (HR = 1.33; 95%CI: 1.01, 1.75. p = 0.045).

After multivariable regression and interaction analysis, the use of statins and gender had a significant effect on BP. Table 2. Statins were found to have a significant interaction on the effect of BP on survival (p int = 0.015). BP was found to be an independent predictor of overall survival in patients without use of statins (HR = 2.07;95%CI: 1.17, 3.67. p = 0.013) but not in patients who were taking statins (HR = 1.08;95%CI: 0.84, 1.39. p = 0.547). No interaction was found with gender. Male patients who received a BP were associated with shorter survival (HR = 1.32;95%CI: 1.68, 1.04. p = 0.021). When the multivariable analysis was restricted to patients who received a pericardium bioprosthesis, the risk was still higher where statins were not used (HR = 2.31;95%CI: 1.25, 4.30).

**Table 2:** Final model for hazard ratio for overall survival in patients with BP after multivariable adjustment (n = 1196).

Variable	HR(95%CI)	p	P for interaction
Bioprosthesis	-	-	0.031
No statins	2.07 (1.17, 3.67)	0.013	
Statins	1.08 (0.84, 1.39)	0.547	
Age	1.01 (0.97, 1.05)	0.712	
Male	1.32 (1.68, 1.04)	0.021	0.362
Creatinine	1.03 (0.94, 1.14)	0.525	
Diabetes	1.25 (0.96, 1.63)	0.102	
Hypertension	1.27 (0.95, 1.70)	0.105	
CABG	1.14 (0.91, 1.44)	0.255	
Obesity	0.83 (0.61, 1.12)	0.221	
EuroSCORE	1.10 (1.07, 1.13)	<0.001	

CABG: Coronary artery bypass grafts.

### Secondary outcomes

Un-adjusted CV survival was shorter in patients who received bioprosthesis (Fig 2). No significant differences were found after adjustment for confounders (HR = 2.60;95%CI: 0.91, 7.45. p = 0.073). No differences in postoperative outcomes were found (Table 3).

**Table 3:** Postoperative outcomes.

	Bioprosthesis (986)	Mechanical (210)	P
Operative mortality	45 (4.6)	12 (5.7)	0.477
Surgical re-exploration	51 (5.2)	10 (4.8)	0.806
Postoperative infections	125 (12.7)	28 (13.3)	0.796
Stroke	24 (2.4)	2 (0.9)	0.181
Atrial Fibrillation	282 (28.6)	63 (30.0)	0.684
AV block	52 (5.3)	10 (4.5)	0.761
PPM	29 (2.9)	5 (2.4)	0.657
Acute renal failure	92 (9.3)	22 (10.5)	0.671

AV: Atrioventricular; PPM: Permanent pacemaker.

Baseline variable comparison between missing and not missing NYHA values are described in S3 Table. Exclusion of cases with missing values in the final model did not modify the main outcomes (S4 Table).

## Discussion

**Fig 3:**
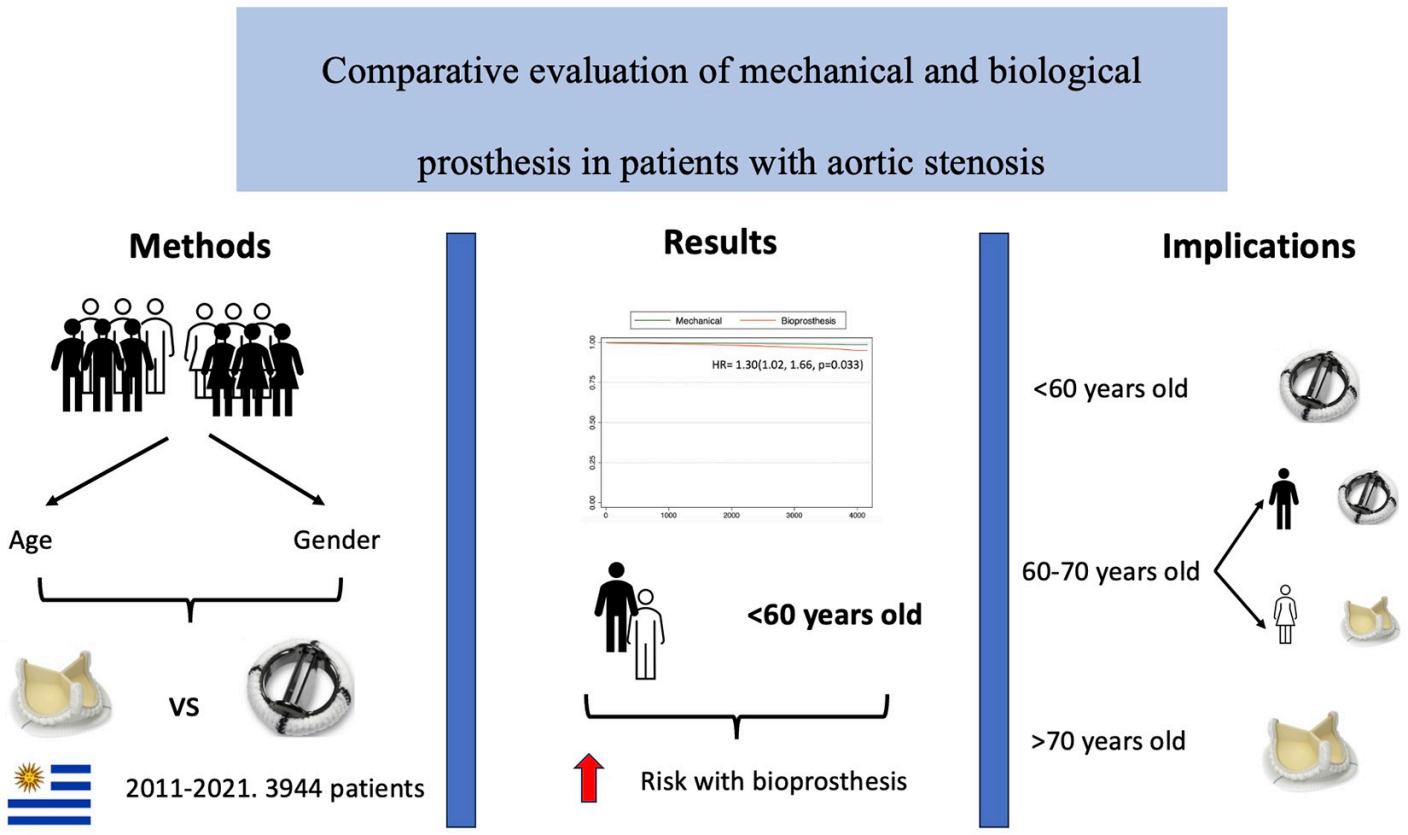
Patients who underwent aortic valve replacement in Uruguay from 2011 to 2021 were included. Interaction was evaluated for use of statins.

After multivariable analyses, BP was associated with poorer survival in male patients and in patients not taking statins (Fig 3).

There is no clear-cut age point at which ACC/AHA or ESC/EACTS guidelines preferentially support BP or MP. It has been recommended that patients younger than 50 years old may benefit from MP and older that 70 years old from BP [1,2]. Further retrospective studies have shown contradictory results, especially in the middle age range spanning from 60–70 years old. Chiang et al in a retrospective 10 year follow-up of SAVR patients in the state of New York have shown no differences on long term mortality in this age group [14]. Similarly, Attia et al using data from the Cleveland Clinic found no differences in long term survival between MP and BP [15]. Additionally, they found no differences in survival among patients who required reoperation. It is likely that data from Attia et al have limited external validity and should be interpreted with caution when applied to non-high volume cardiac surgery centers or population-level decisions. In contrast, other authors such as Brown et al [16] in the United Stated and Glaser et al [17] in Sweden have shown improved survival in this age group with MP. These same authors, using data from SWEDEHEART have found that survival with the Carpentier-Edwards PERIMOUNT valve (Edwards Lifesciences Corp) was better than other bioprosthesis but not better than with MP [18].

Most of the data regarding MP vs BP come from the US or Europe. Management of anticoagulation as well as technical expertise in cardiac surgery centers have an evident impact in the relative benefit of MP or BP. Therefore, evidence from centers outside US and Europe is needed to inform a global understanding of the question. The main dis-advantages of BP are degeneration and re-operation. If severe degeneration is detected in a timely manner, and re-operation performed successfully, its disadvantages may be reduced and the turning point in age shifts to younger patients.

Evidently the solution to the best prosthesis in the 60–70 year old range is not absolute and there is a need to identify sub-group of patients which may benefit with either BP and MP. Gender is an important sub-group which unfortunately has been scarcely evaluated in the literature. Kulik et al have found better outcomes after BP in female patients [5] probably due to lower need of re-operations. A reduced need for re-operation in female patients after SAVR was also demonstrated in a Nationwide study in Finland [6]. Hammermeister et al in a RCT of male patients found greater long term mortality associated with BP [7]. Other studies have shown gender to be a strong confounder in the relative benefit of MP [8]. We have shown that gender has a strong effect in outcomes after SAVR and should be seriously considered in 60–70 year old patients. Our data suggest that in this age range, male patients should probably receive MP while female patients may benefit from either strategy.

Although high dose statin therapy has failed to stop native aortic valve calcification [9], there is scarce evidence on its benefit in BP. Antonini-Canterini et al. have been shown an association between statin use and reduction in the progression of BP degeneration [10]. Rosuvastatin has been used in animal models and shown to decrease BP calcification [11]. Additionally, evidence from Swedish registries have shown association between statin use and survival in patients after SAVR [12,13]. In our cohort, patients who received BP and were under statins had better outcomes. Although adherence to medication after SAVR use was not a variable available in our database, probably these patients were kept under statins due to concomitant dyslipidemia and the benefit was driven mainly due to its reported role in slowing down BP degeneration. Further randomized control trials are required to test this association.

### Limitations

As a retrospective study, selection bias is the main limitation. Adherence to statin treatment was not evaluated due to lack of centralized drug dispatch orders as well as lack of individual follow-up of patients. In order to adjust for measured confounders we performed Cox regression analysis. Nonetheless, we acknowledge the existence of un-measured confounders which may affect the outcome.

We used the National database to extract outcome variables. Follow-up of post-operative outcomes or cross-link with other National databases is suboptimal and therefore we were not able to evaluate important outcomes such as: re-admission, re-operation or stroke, bleeding. Additionally, we were not able to address the postoperative management (use of antiaggregation and anticoagulation) of patients since this information is not included in the mandatory registry required by the National Agency. Cause of death was recorded by the Ministry of Health and extracted from the death certificate which may not be completely accurate.

Due to financial restrictions, we are not able to use pericardial prosthesis with better long-term durability and many of our patients have received bioprosthesis which have been withdrawn from the market. Pericardial prosthesis with better long-term outcomes such as Perimount and Avalus were used in very few patients (64 patients in total) which does not allow us to perform a subgroup analysis including only these patients.

Finally, we would like to highlight the inherent limitation due to the number of patients included which may explain the different outcome according to gender (lower amount of female patients).

## Supporting information

S1 Table Type of prosthesis. (DOCX)

S2 Table Univariate predictors of survival. (DOCX)

S3 Table Baseline variable comparison between missing and not missing NYHA values. (DOCX)

S4 Table Final model for hazard ratio for overall survival in patients with BP after multivariate adjustment with exclusion of patients with missing values (n = 1017). (DOCX)

## Abbreviations

BP: Bioprosthesis
CABG: Coronary artery bypass grafts
EF: Ejection fraction
FNR: Fondo Nacional de Recursos
HTN: Hypertension
IRR: Incidence rate ratio
MP: Mechanical prosthesis
RCT: Randomized control trial
SAVR: Aortic valve replacement
TAVI: Transaortic valve implantation
