# Supplementary material for: Medium-term survival of patients with mechanical and biological aortic prosthesis at the 6th decade of life
Source: PLoS One. 2024 Nov 18;19(11):e0312408. doi: 10.1371/journal.pone.0312408 (PMC11573135; doi:10.1371/journal.pone.0312408)
Supplement: S1 Table — (DOCX) [file pone.0312408.s001.docx]

S1 Table. Type of prosthesis

| Prosthesis Type |  |
| --- | --- |
| Braille | 14 (1.17) |
| Carpentier-Edwards | 59 (4.93) |
| Dokimo | 25 (2.09) |
| Avalus | 5 (0.42) |
| Hancock I | 77 (6.44) |
| Hancock II | 46 (3.85) |
| Mosaic | 142 (11.87) |
| Crown | 113 (9.45) |
| Mitroflow | 226 (18.90) |
| Perceval | 6 (0.50) |
| St Jude - Epic | 273 (22.83) |
| ATS Medical - AP | 4 (0.33) |
| Cryolife - ONX | 16 (1.34) |
| Sorin - Bicarbon | 95 (7.94) |
| St Jude - Masters | 74 (6.19) |
