# Supplementary material for: Medium-term survival of patients with mechanical and biological aortic prosthesis at the 6th decade of life
Source: PLoS One. 2024 Nov 18;19(11):e0312408. doi: 10.1371/journal.pone.0312408 (PMC11573135; doi:10.1371/journal.pone.0312408)
Supplement: S2 Table — (DOCX) [file pone.0312408.s002.docx]

S2 Table. Univariate predictors of survival.

| Variable | Univariate (HR95%CI) | p |
| --- | --- | --- |
| Bioprosthesis | 1.46 (1.01, 2.11) | 0.042 |
| Age | 1.06 (1.02, 1.10) | 0.002 |
| Female | 0.83 (0.66, 1.05) | 0.121 |
| Creatinine | 1.16 (1.06, 1.27) | 0.001 |
| Dyslipidemia | 1.28 (1.03, 1.60) | 0.026 |
| Diabetes | 1.39 (1.09, 1.77) | 0.008 |
| Hypertension | 1.33 (1.01, 1.75) | 0.045 |
| CABG | 1.19 (0.94, 1.49) | 0.143 |
| Euroscore | 1.10 (1.08, 1.13) | <0.001 |
