# Supplementary material for: Medium-term survival of patients with mechanical and biological aortic prosthesis at the 6th decade of life
Source: PLoS One. 2024 Nov 18;19(11):e0312408. doi: 10.1371/journal.pone.0312408 (PMC11573135; doi:10.1371/journal.pone.0312408)
Supplement: S3 Table — (DOCX) [file pone.0312408.s003.docx]

S3 Table. Baseline variable comparison between missing and not missing NYHA values.

|  | Not missing values (1017) | Missing values (179) | p |
| --- | --- | --- | --- |
| Age (SD) | 66.0 (3.1) | 66.1 (2.9) | 0.722 |
| Female (%) | 406 (39.9) | 62 (34.6) | 0.182 |
| HTN (%) | 788 (77.5) | 140 (78.2) | 0.882 |
| Diabetes (%) | 243 (23.9) | 40 (22.3) | 0.653 |
| Smoker (%) | 226 (22.2) | 48 (26.8) | 0.178 |
| Obesity (%) | 176 (17.3) | 28 (15.6) | 0.585 |
| Creatininemia (mg/dl) | 1.08 (0.82) | 0.97 (0.24) | 0.075 |
| Use of statins (%) | 401 (39.4) | 73 (40.8) | 0.733 |
| Previous MI (%) | 4 (0.39) | 15 (8.4) | <0.001 |
| AF (%) | 12 (1.2) | 19 (10.6) | <0.001 |
| LVEF (SD) | 57.5 (10.5) | 58.3 (10.7) | 0.348 |
| Concomitant CABG (%) | 324 (31.2) | 47 (26.3) | 0.135 |
| EuroSCORE (SD) | 4.9 (3.0) | 4.6 (2.0) | 0.173 |

HTN: hypertension; NYHA: New York heart association; LVEF: left ventricular ejection function; MI: myocardial infarction.
