# Supplementary material for: Medium-term survival of patients with mechanical and biological aortic prosthesis at the 6th decade of life
Source: PLoS One. 2024 Nov 18;19(11):e0312408. doi: 10.1371/journal.pone.0312408 (PMC11573135; doi:10.1371/journal.pone.0312408)
Supplement: S4 Table — (DOCX) [file pone.0312408.s004.docx]

S4 Table. Final model for hazard ratio for overall survival in patients with BP after multivariate adjustment with exclusion of patients with missing values (n=1017)

| Variable | HR(95%CI) | p | P for interaction |
| --- | --- | --- | --- |
| Bioprosthesis |  |  | 0.045 |
| No statins | 2.68 (1.32, 5.45) | 0.006 |  |
| Statins | 1.09 (0.83, 1.43) | 0.529 |  |
| Age | 1.01 (0.97, 1.06) | 0.658 |  |
| Male | 1.38 (1.78, 1.07) | 0.015 | 0.565 |
| Creatinine | 1.01 (0.91, 1.12) | 0.818 |  |
| Diabetes | 1.18 (0.88, 1.57) | 0.267 |  |
| Hypertension | 1.46 (1.06, 2.03) | 0.021 |  |
| CABG | 1.09 (0.84, 1.41) | 0.508 |  |
| Obesity | 0.88 (0.64, 1.22) | 0.444 |  |
| EuroSCORE | 1.11 (1.08, 1.15) | <0.001 |  |
